# Emergent surgical removal of a migrated atrial septal defect occluder: case report

**DOI:** 10.1186/s13019-020-01350-5

**Published:** 2020-11-11

**Authors:** Bi Wen, Juan He

**Affiliations:** Department of Cardiology, West China Hospital, Sichuan University, Guoxuexiang 37th, 610041 Chengdu, Sichuan P.R. China

**Keywords:** Surgical retrieval, ASD closure device, Left ventricular outflow tract

## Abstract

**Background:**

Atrial septal defect (ASD) closure has been widely accepted and is now routinely performed using a percutaneous approach under especially echocardiographic guidance Transesophageal echocardiography (TEE). One major complication is dislocation of occluder device during or after the device implantation. Surgical removal may be required, especially when the device stuck in the left ventricular outflow tract (LVOT).

**Case introduction:**

A 21-year-old female was admitted to our department for percutaneous closure of secundum ASD. Percutaneous closure under the guidance of TEE was recommended for the patients. During device implantation, the TEE showed dislocation of the 22 mm ASD occluder device, stucked into the LVOT and behind the anterior mitral leaflet, producing severe LVOT obstruction Fig. 1). We herein present a safe and quick technique for surgical removal of an ASD occlude device located in the LVOT.

**Conclusion:**

This technique provides a safe method for surgical removal of malposition and migration ASD occluder device.

## Background

Atrial septal defect (ASD) is one of the most common congenital anomalies. Percutaneous closure of secundum ASD has evolved over the past three decades, and is considered a standard treatment for ASD in recent years [1]. Although infrequent, complications such as arrhythmia, embolization, thrombosis, dislocation of occluder device and perforation have been reported associated with this procedure [2]. The occluder device be retrieved percutaneously sometimes, however surgical removal may be needed, especially when the device stuck in the LVOT and left atrial disk towards the ASD. It is difficult to remove the device without damage to the chordae and mitral valve. We present a safe and quick technique for surgical removal of ASD closure device stuck in the LVOT behind the anterior mitral leaflet.

## Case report

**Fig. 1:**
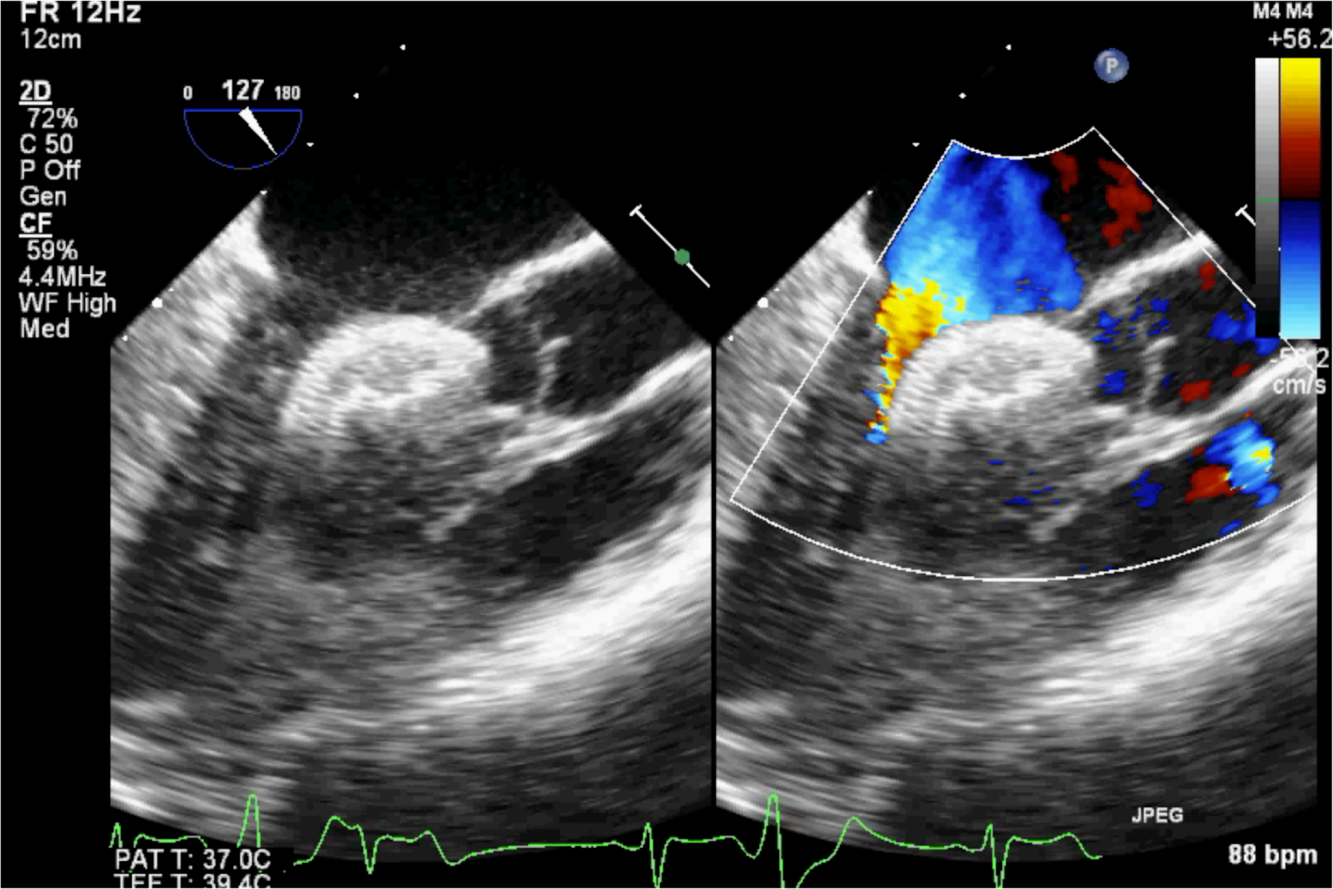
TEE showed the ASD occluder device trapped in into the LVOT and behind the anterior mitral leaflet, producing severe LVOT obstruction


A 21-year-old female was admitted to our department for percutaneous closure of secundum ASD because of progressive decrease in exercise tolerance. Transthoracic echocardiography (TTE) revealed a 16 mm secundum ASD with adequate rims, the posterior rim, superior rim, inferior rim were 10 mm, 12 mm, 16 mm respectively. Left to right shunt and moderate right-sided heart enlargement were also proved by TTE. Percutaneous closure under the guidance of TEE with 22 mm ASD occluder device was recommended for the patients. During device implantation, the TEE showed dislocation of the 22 mm ASD occluder device (Shanghai Shape Memory Alloy, China), stucked into the LVOT and behind the anterior mitral leaflet, producing severe LVOT obstruction (Fig. 1, videos 1 and 2).

**Additional file 1: Video 1.**


**Additional file 2: Video 2.**

**Fig. 2:**
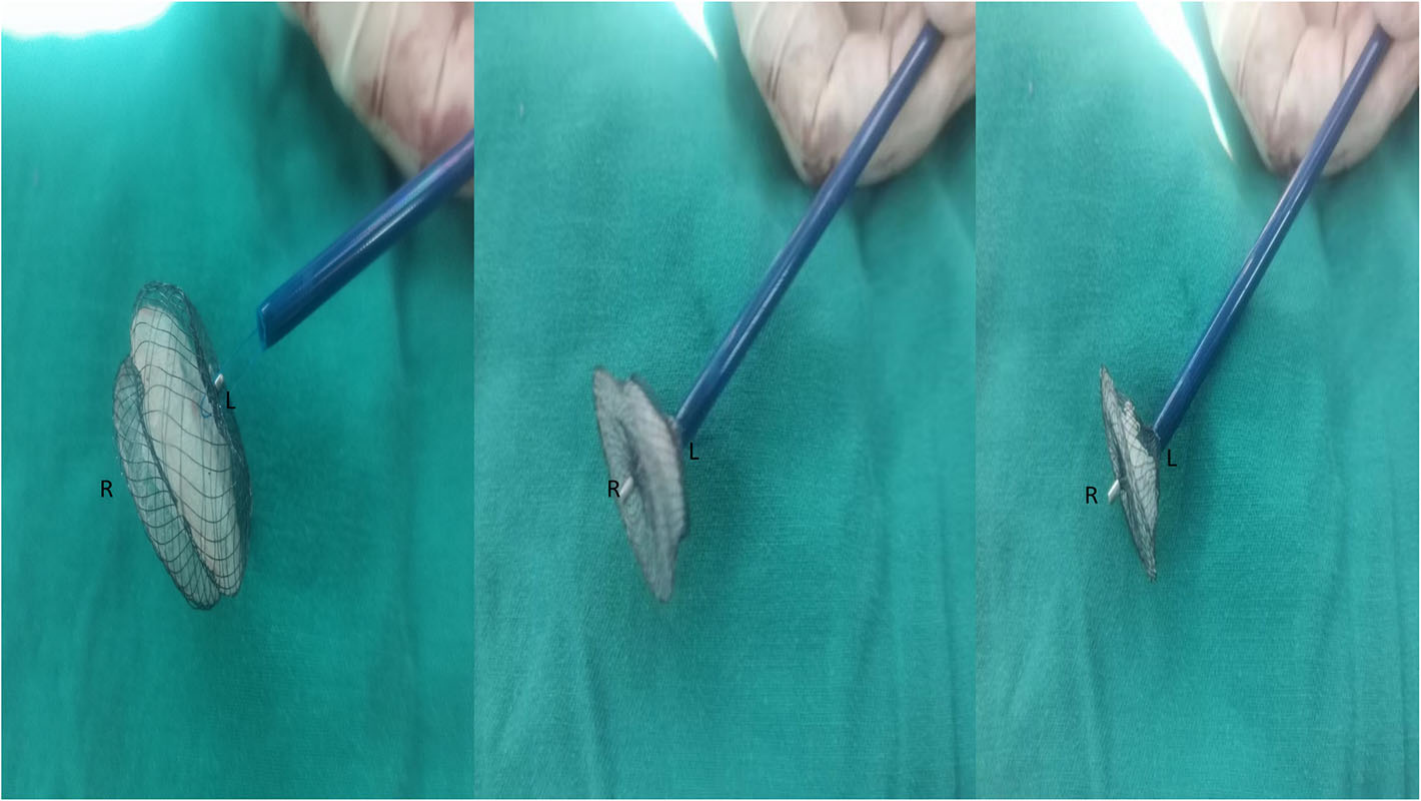
Retrieval of ASD occluder device into a snugger to facilitate safe removal from the LVOT when the left atrial disk towards the ASD

Emergent surgical removal of the ASD occluder device was performed immediately with general anesthesia. A standard median sternotomy was performed in the patient, after institution of cardiopulmonary bypass, The ASD was approached via right atriotomy. The ASD occluder device was stuck in the LVOT, behind the anterior mitral leaflet, twined by the mitral chordae and the left atrial disk towards the ASD, making it difficult to hold the device by its screw located on the right atrial disk. So 3/0 prolene suture is placed through the middle of the device in the right disk of the occluder device. Then the 3/0 prolene suture is passed through a 16F soft plastic snugger (Shanghai Shape Memory Alloy, China) which is advanced well into the ASD avoiding of any chordal apparatus. As the suture is pulled, the ASD occlude device is gradually retrieved into the snugger. Finally, the snugger can be removed from the heart safely with the compressed device inside (Fig. 2), then the ASD was closed with pericardial patch. The postoperative course was uneventful.

## Discussion

Recently improvements in device design and operator experience have resulted in increasing adoption of percutaneous ASD closure, complications such as arrhythmia, embolization, thrombosis, dislocation of occluder device and perforation have been reported associated with this procedure [2, 3]. Device malposition and migration are one of the major complications following ASD device closure, often requiring emergent or urgent surgical intervention [4]. AND TEE is the important means to get the exact morphology of the ASD, such as the size, position in the interatrial septum, and adequacy of septal rims [5, 6].

Although successful percutaneous retrieval of the device are widely reported in the literature, surgical removal was necessary when the occluder devices are stucked in the special location [5]. Device dislocation generally occur early and may be related to the initial positioning of this device [4], the rim was inadequate especially when retro-aortic rim less than 5 mm [6]. The device dislocation reason of our case is the device cable connecting with the disk was loose during the deployment of the left-sided disk.

Various techniques for surgical removal of closure devices have been reported such as direct retrieval of the deployed device. Such devices are deployed from a sheath and when being removed percutaneously are resheathed for safe removal [7]. However, the authors only described the technique applicable to right atrial disk towards the ASD. Actually, when the left disk of the occluder device towards the ASD and twined by the mitral chordae, the technique also worked. Our technique keep the surgical removal quick and safe and minimize valve damage and conduction system complications. It is important to understand the technique for any cardiac surgeon involved in the care of such patients [7].

## Conclusion

This technique provides a safe method for surgical removal of malposition and migration ASD occluder device.

## Data Availability

The datasets used are available from the corresponding author on reasonable request.

## Abbreviations

ASD: Atrial septal defect
TEE: Transesophageal echocardiography
LVOT: Left ventricular outflow tract
LV: Left ventricle
LA: Left atrium
AO: Aorta
